# Supplementary material for: Adaptive Thermogenesis and Lipid Metabolism Modulation in Inguinal and Perirenal Adipose Tissues of Hezuo Pigs in Response to Low-Temperature Exposure
Source: Cells. 2025 Mar 7;14(6):392. doi: 10.3390/cells14060392 (PMC11941736; doi:10.3390/cells14060392)
Supplement: Supplementary file 1 [file cells-14-00392-s001.zip › cells-3389046-supplementary.pdf]

Supplementary Materials:

Table S1. Sequencing data quality control. A: Low temperature treatment for 24 h; B: Low temperature treatment for 5 d; C：Low temperature treatment for 10 d.

Group	Raw reads	Clean reads	clean ratio (%)	Q20 (%)	Q30 (%)	GC content (%)
A_NPF	53062086	50699823	95.52	98.89	96.98	49.19
A_CPF	51112111	49337538	96.52	98.72	96.58	49.77
B_NPF	48109091	46971630	97.64	98.80	96.75	49.32
B_CPF	48395665	47132484	97.36	98.71	96.59	48.85
C_NPF	48486741	47228114	97.40	98.76	96.65	50.38
C_CPF	47911099	46564100	97.19	98.71	96.55	49.87
A_NIF	51882524	50647287	97.60	98.67	96.45	50.93
A_CIF	51406099	50020545	97.30	98.75	96.66	48.76
B_NIF	48703935	46726276	95.93	98.71	96.48	52.09
B_CIF	47656243	46316375	97.20	98.83	96.76	49.6
C_NIF	49616929	48267540	97.28	98.82	96.78	48.73
C_CIF	51731764	50020950	96.79	98.85	96.83	48.66

Table S2. Summary of RNA-seq alignment. A: Low temperature treatment for 24 h; B: Low temperature treatment for 5 d; C：Low temperature treatment for 10 d.

Group	Total sequenced reads	Total aligned reads	Mapping Ratio	Uniquely aligned reads	Multiple aligned reads
A_NPF	50699823	48192544	95.08%	92.75%	2.33%
A_CPF	49337538	46848972	94.97%	92.85%	2.12%
B_NPF	46971630	44876211	95.54%	93.47%	2.08%
B_CPF	47132484	44778525	95.01%	93.08%	1.93%
C_NPF	47228114	45014756	95.32%	92.77%	2.54%
C_CPF	46564100	44353295	95.25%	92.83%	2.42%
A_NIF	50647287	48249581	95.29%	92.62%	2.67%
A_CIF	50020545	47553382	95.08%	93.21%	1.87%
B_NIF	46726276	43951239	94.02%	91.51%	2.52%
B_CIF	46316375	44249061	95.54%	93.39%	2.15%
C_NIF	48267540	46186737	95.70%	93.34%	2.35%
C_CIF	50020950	47840014	95.66%	93.46%	2.20%
